# Regional Colon and Rectal Surgeon Density Is Associated with Variation in Rectal Cancer Surgical Treatment: A Dartmouth Atlas Study

**DOI:** 10.3390/jcm14062004

**Published:** 2025-03-15

**Authors:** Srinivas J. Ivatury, Daniel L. Underbakke, Ravinder Kang

**Affiliations:** 1 Department of Surgery and Perioperative Care, University of Texas at Austin Dell Medical School, 1601 Trinity Street Building B, Austin, TX 78712, USA; 2 Department of Surgery, Dartmouth-Hitchcock Medical Center, Lebanon, NH 03766, USA

**Keywords:** variation, rectal cancer, specialization, Medicare

## Abstract

**Background/Objectives**: Recent reports reflect the increased enthusiasm for restorative reconstruction after a proctectomy (LAR) for rectal cancer in appropriate candidates. Despite this, abdominoperineal resection (APR) remains common. We aimed to examine the effect of the colorectal surgeon density in a hospital referral region (HRR) on the rates of LARs and APRs performed. **Methods**: We conducted a retrospective cohort study of Medicare-participating hospitals in the United States for the fiscal year 2014. Our cohort was all Medicare beneficiaries (MBs) with rectal cancer (ICD-9: 154.1) who underwent an intervention of an LAR (CPT: 44145, 44146, 44207, 44298, 45112, 45397) or an APR (CPT: 45110 or 45395). We compared the APR and LAR rates per HRR with the density of board-certified colorectal surgeons per HRR (divided into low-, medium-, and high-density HRRs) using membership and zip code data from the American Board of Colon and Rectal Surgery. **Results**: A total of 3366 beneficiaries underwent LARs and 1821 beneficiaries underwent APRs for rectal cancer in 2014. The national rates of LARs and APRs were 12.12 and 6.66 per 100,000 MBs, respectively. The individual rates were available for 104 HRRs for the LARs and 46 HRRs for the APRs (those with >10 procedures/year). The median rates of LARs per 100,000 MBs in the low-, medium-, and high-density groups were 12.13, 13.05, and 14.25, respectively. The median rates of APRs per 100,000 MBs in the low-, medium-, and high-density groups were 7.69, 7.29, and 6.23, respectively. Both trends were significant by a test of trend. **Conclusions**: A higher colorectal surgeon density was associated with increased rates of LARs and decreased rates of APRs for Medicare beneficiaries.

## 1. Introduction

Advancements in surgical technique and multimodal therapy for rectal cancer has allowed more patients to be candidates for restorative reconstruction with a low anterior resection (LAR) [1,2,3,4,5]. Despite these improvements, abdominoperineal resection (APR) remains the predominant surgical approach in many regions of the United States, even for those patients who could be considered for an LAR.

This observation suggests that a disparity in treatment strategies exists based on geography, leading to patients with similar clinical characteristics receiving a permanent colostomy in one region and a restorative reconstruction in another. Geographic disparities in treatment was shown in prior work that demonstrated the variation in surgical therapy for breast cancer, where patients in urban centers and those that underwent care at large institutions were more likely to undergo breast conservation than a mastectomy [6]. Similar variations were observed for several other disease processes across medical specialties, including chronic kidney disease, post myocardial infarction cardiac care, cesarian births, and gastric cancer treatment [7,8,9,10].

Potential factors driving geographic variability may include the increased complexity of disease, referral patterns, or case mix index differences. The specialist surgeon availability may also contribute; the availability of pediatric surgery specialists is associated with a decreased incidence of bowel resection during the treatment of intestinal intussusception, and the overall outcomes in pediatric surgical care are directly correlated with the degree of specialization of the operating surgeon [11,12]. The effect of specialist surgeon availability on the LAR and APR rates is unknown.

Colon and rectal surgeons are specialist surgeons for rectal cancer. Colon and rectal surgeons are specifically trained in minimally invasive techniques, the management of locally advanced and complex rectal cancer cases, and the multimodal treatment of rectal cancer in concert with gastrointestinal oncologists and radiation oncologists. Colon and rectal surgical training allows for colon and rectal surgeons to offer reconstructive surgical options for rectal cancer that may not be offered by surgeons with general surgical training alone, impacting the options available for surgical decision making. Additionally, colon and rectal surgeons often lead multidisciplinary cancer teams for rectal cancer, which also impacts the treatment and surgical choices. An increasing colon and rectal surgeon regional density likely results from the development of colon and rectal surgical practices, while a lower density suggests solo practice or colon and rectal surgeons joining multispecialty groups with general surgeons and other surgical specialists. This may impact the number and acuity of patients with rectal cancer exclusively treated by specialist colon and rectal surgeons compared with general surgeons and other surgical specialties in a region.

The hypothesis of the current study is that a higher regional colon and rectal surgeon density is associated with higher rates of LARs and lower rates of APRs. Therefore, the goal of this study was to evaluate the influence of the board-certified colon and rectal surgeon density in hospital referral regions (HRRs) across the United States on the rates of LARs and APRs in a population of Medicare beneficiaries with rectal cancer.

## 2. Materials and Methods

We used the Dartmouth Atlas Rate Generator of The Dartmouth Atlas of Health Care to view region-specific data from the 306 hospital referral regions (HRRs) in the United States [13]. The Dartmouth Atlas Rate Generator is a research tool used to view region-specific data and perform comparisons and analysis of the regions within the United States healthcare system using Medicare claims. Hospital referral regions represent regional healthcare markets for tertiary medical care that generally require the services of a major referral center.

Our dataset was the 2014 MedPAR data file, the most currently available cohort of Medicare beneficiaries. This data file contained 100% Medicare Part B Claims Data for the fiscal year 2014. All beneficiaries with an ICD-9 code for rectal cancer (154.1) who underwent LARs or APRs during the calendar year 2014 were included. The APR group included those beneficiaries with the following CPT codes: 45110 or 45395. The LAR group included those beneficiaries with the following CPT codes: 44145, 44146, 44207, 44298, 45112, and 45397. The codes for restorative LAR procedures were selected by consensus of the authors and experts for appropriateness for inclusion [14].

Using the Dartmouth Atlas Rate Generator, we estimated the rates of LARs and APRs per beneficiary in each of the 306 HRRs. We calculated the rates for each procedure per 100,000 Medicare beneficiaries in the HRRs where 11 or more of the specific procedure were performed in 2014. We excluded those HRRs with less than 11 from the final analysis due to the Dartmouth Atlas data use agreement.

**Figure 1:**
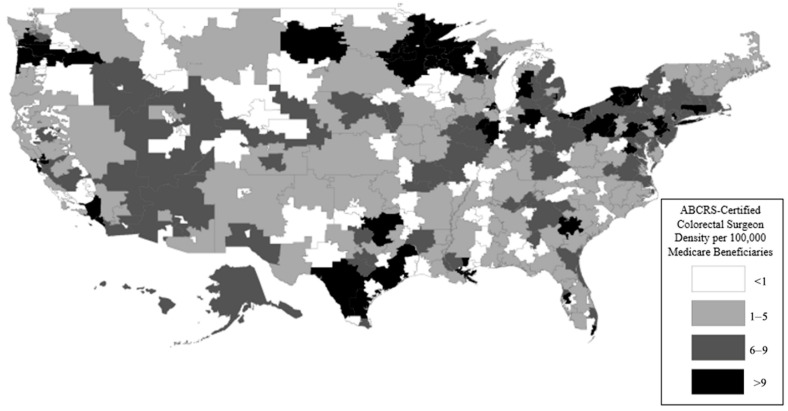
ABCRS-certified colorectal surgeon density per 100,000 Medicare beneficiaries. This figure demonstrates that there was a wide variation in the concentration of ABCRS-certified colorectal surgeons in each hospital referral region. Specifically, there was a higher density in metro area HRRs (smaller-sized HRRs), as well as HRRs in the northern, southern, and coastal parts of the United States.

We grouped the HRRs by the density of active, board-certified colon and rectal surgeons in 2014 using certification data provided by the American Board of Colon and Rectal Surgery (ABCRS) active roster of diplomates; this active roster from 2015 provided addresses and certification data for each member. We excluded the 88 surgeons that were certified in 2015 or not practicing in the United States, which left 898 active, ABCRS-certified colon and rectal surgeons that were practicing in the United States. We assigned members to their respective HRRs by the 2014 Dartmouth Atlas zip code to the HRR crosswalk file, which associates zip codes with their respective HRRs (Figure 1).

The colorectal surgeon density for each HRR was calculated using the number of colorectal surgeons divided by the total number of Medicare beneficiaries in each HRR. The HRRs were divided into three categories for comparison based on the colorectal surgeon density: low density—less than five colorectal surgeons per 100,000 Medicare beneficiaries, medium density—five to nine, and high density—greater that nine surgeons. We chose this breakdown following previously published rates of general surgeons per 100,000 beneficiaries in The Dartmouth Atlas of Healthcare. The average rates of surgical specialists per 100,000 Medicare beneficiaries per HRR was 50 specialists; each of the density groups constituted less than 10%, 10–20%, and greater than 20% of all surgical specialists per 100,000 Medicare beneficiaries per HRR. We identified these percentages based on previous Atlas analyses and with consultation from the experts at the Dartmouth Atlas project.

We then compared the rates of APRs and LARs by surgeon density category. We calculated the coefficients of variance for each density cohort and a test of the trend on each surgical procedure using STATA 15 (StataCorp LLC—College Station, TX, USA). The density maps by HRR were created using ArcMap 10.6 (Esri Inc.—Redlands, CA, USA).

## 3. Results

**Figure 2:**
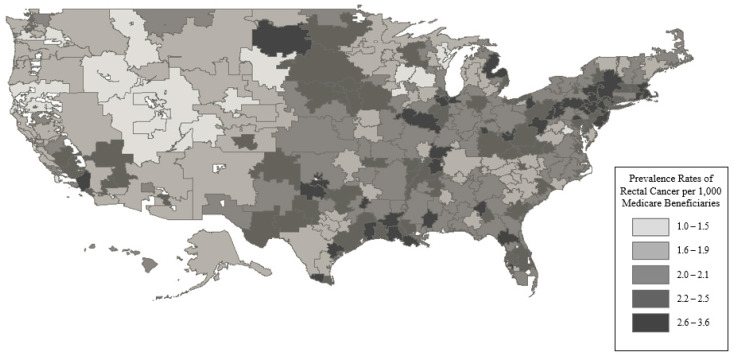
Prevalence rates of rectal cancer per 1000 Medicare beneficiaries. This figure demonstrates that there was an absolute difference in the rectal cancer prevalence of around 3 cases per 1000 Medicare beneficiaries between the lowest and highest quintile groups. This suggests that there was not a large variation by region.

The prevalence of rectal cancer was 57,499 in the 27,767,493 Medicare beneficiaries (MBs) in 2014. The rates of rectal cancer ranged from 1.01 to 3.56 per 1000 MBs, as illustrated in Figure 2.

**Figure 3:**
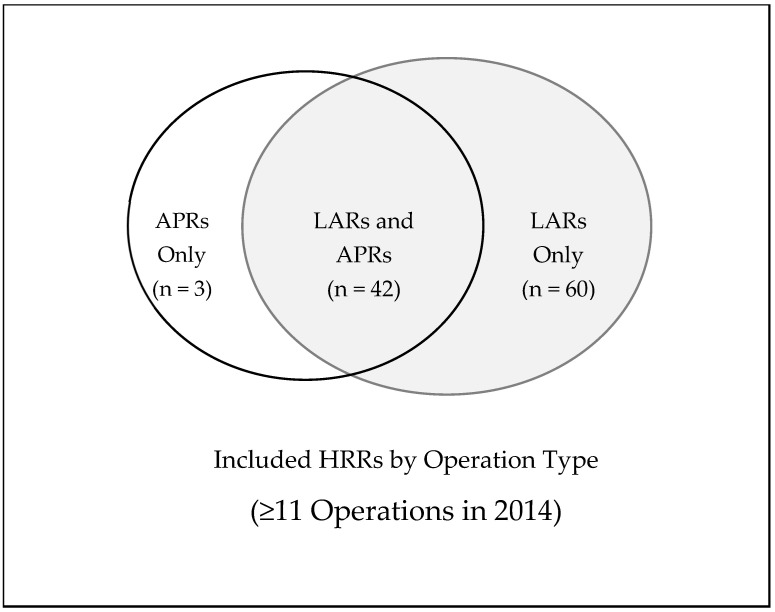
Graphical representation of the HRRs included in the analysis by operation type. Of the 306 HRRs in the United States, there were 105 HRRs (34%) that performed 11 operations or greater on Medicare beneficiaries in 2014. This demonstrates that most HRRs had less than 11 cases of major rectal cancer surgery performed on Medicare beneficiaries. These data suggest that most major rectal cancer surgeries were performed in low-volume centers.

**Figure 4:**
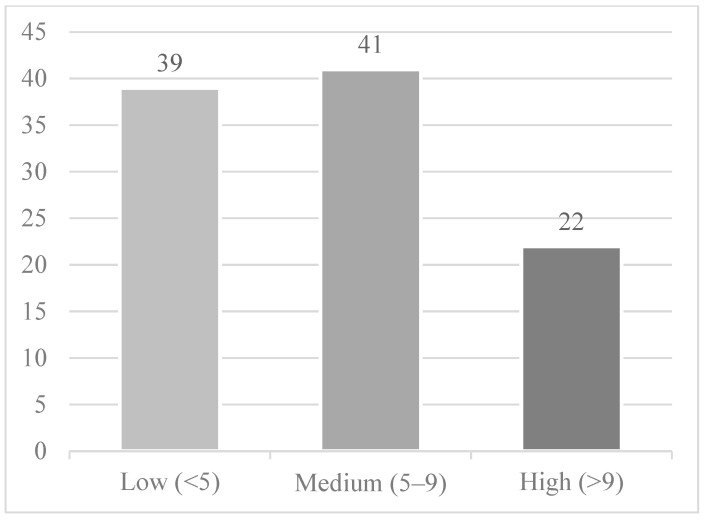
Hospital referral regions by ABCRS-certified colon and rectal surgeon density per 100,000 Medicare beneficiaries included for the LAR analysis (the remaining 202 HRRs were suppressed due to the data use agreement). This figure demonstrates that the majority of HRRs that performed at least 11 LAR operations in 2014 had either a low or medium density of ABCRS-certified colorectal surgeons.

**Figure 5:**
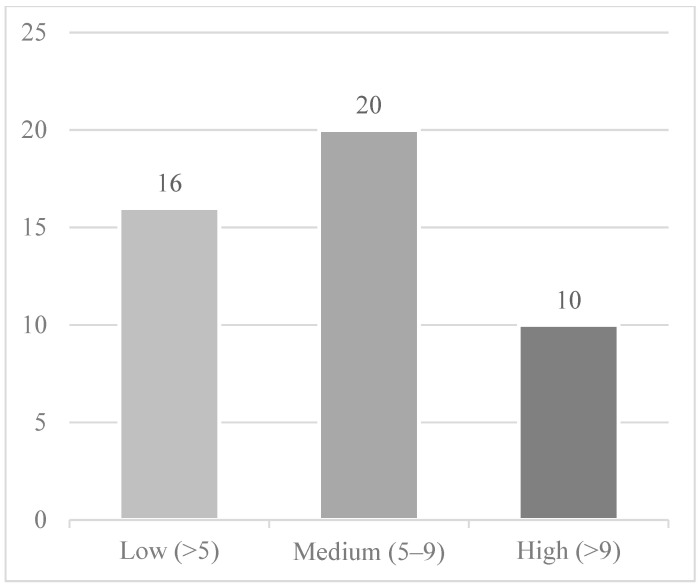
Hospital referral regions by ABCRS-certified colon and rectal surgeon density per 100,000 Medicare beneficiaries included for the APR analysis (the remaining 260 HRRs were suppressed due to the data use agreement). This figure demonstrates that the majority of HRRs that performed at least 11 APR operations in 2014 had either a low or medium density of ABCRS-certified colorectal surgeons.

A total of 3366 MBs underwent LARs and 1821 MBs underwent APRs for rectal cancer in 2014, which accounted for 9.0% of the total MBs with rectal cancer in 2014. The national rates of LARs and APRs were 12.12 and 6.66 per 100,000 MBs, respectively. A total of 102 HRRs for LARs had HRR level rates (those with >10 procedures/year), with a breakdown by colorectal surgeon density as follows: low density—39 HRRs, medium density—41 HRRs, high density—22 HRRs. A total of 46 HRRs for APRs had HRR level rates with the following breakdown by surgeon density: low density—16 HRRs, medium density—20 HRRs, high density—10 HRRs. Figure 3, Figure 4 and Figure 5 display this graphically. These represent 34% and 15% of the total HRRs for LARs and APRs, respectively. In total, 70% of the total LARs and 42% of the total APRs performed in 2014 on MBs were performed in the HRRs included in the analysis.

**Figure 6:**
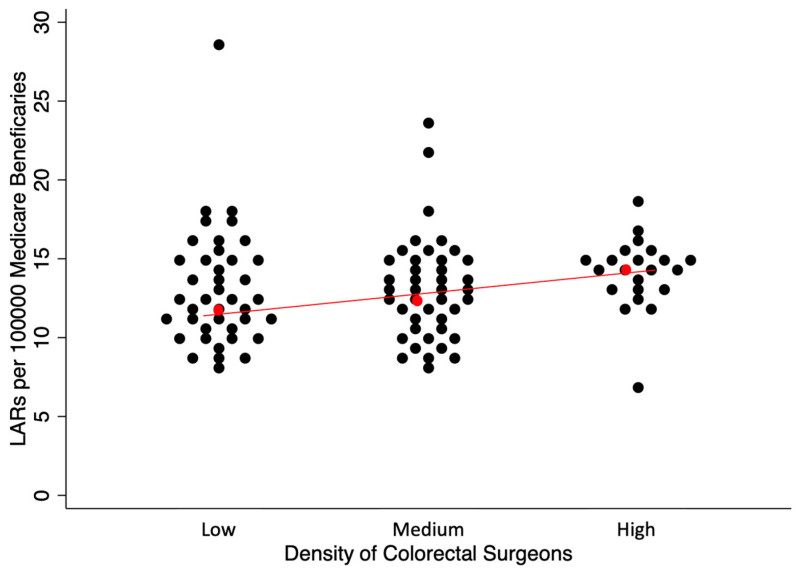
Rates of LARs per 100,000 Medicare beneficiaries by HRRs differentiated by the density of ABCRS-certified colon and rectal surgeons. Each dot represents an HRR that performed at least 11 LAR operations in 2014 in the turnip plot. Red dot represents the median HRR rate with the red line showing the general trend based on density of colorectal surgeons. This figure demonstrates that with the increasing density of ABCRS-certified colon and rectal surgeons, there was an increase in the rate of LARs per 100,000 Medicare beneficiaries. In addition, there was decreased variability in the rates of LARs per 100,000 Medicare beneficiaries due to tighter clustering of the dots as the colorectal surgeon density increased.

**Figure 7:**
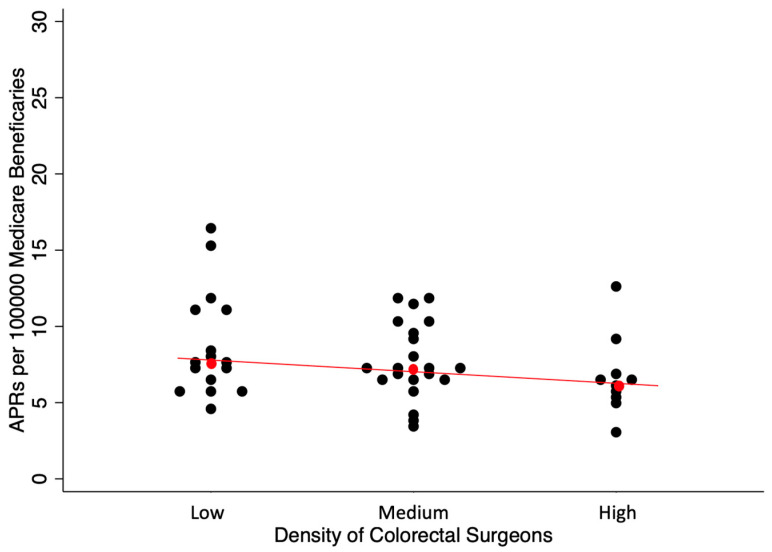
Rates of APRs per 100,000 Medicare beneficiaries by HRRs differentiated by the density of ABCRS-certified colon and rectal surgeons. Each dot represents an HRR that performed at least 11 APR operations in 2014 in the turnip plot. Red dot represents the median HRR rate with the red line showing the general trend based on density of colorectal surgeons. This figure demonstrates that with the increasing density of ABCRS-certified colon and rectal surgeons, there was a decrease in the rate of APRs per 100,000 Medicare beneficiaries. In addition, there was a decreased variability in the rates of APRs per 100,000 Medicare beneficiaries due to the tighter clustering of the dots as the colorectal surgeon density increased.

Figure 6 and Figure 7 demonstrate the distribution of rates for LARs and APRs, and the trend of lower rates of APRs and higher rates of LARs with a higher colorectal surgeon density; these trends were both significant by a test of trend (*p* < 0.001). The median rates of LARs in the low-, medium-, and high-density cohorts were 12.13, 13.05, and 14.25 per 100,000 beneficiaries, respectively. The coefficients of variance for LARs in the low-, medium-, and high-density cohorts were 0.29, 0.25, and 0.16, respectively. The median rates of APRs in the low, medium, and high groups were 7.69, 7.29, and 6.23 per 100,000 beneficiaries, respectively. The coefficients of variance for the APRs in the low-, medium-, and high-density cohorts were 0.39, 0.32, and 0.38, respectively.

## 4. Discussion

LARs were more commonly performed than APRs for Medicare beneficiaries with rectal cancer in 2014. We observed an association of higher rates of LARs and lower rates of APRs with higher colorectal surgeon density, despite the low variability in the prevalence rates of rectal cancer across the HRRs. Only the HRRs with at least 11 procedures performed in 2014 were included in the analysis. We found that 34% of the HRRs included in analysis performed 72% of the total LARs on Medicare beneficiaries with rectal cancer in 2014. For the APRs, however, 15% of the HRRs were included in the analysis and accounted for only 42% of the total APRs. Lastly, we identified less intragroup variation for the LAR rates with higher colorectal surgeon density, while there was no difference in the intragroup variation for the APR rates.

To be included in this LAR or APR analysis, the HRRs must have performed at least 11 of the respective procedure due to data use agreement limitations. In 2014, most LARs were performed in a small proportion of the HRRs. Conversely for the APRs, the 15% of HRRs included accounted for less than half of the APRs performed. This suggests that most LARs for rectal cancer in Medicare beneficiaries were performed in higher-volume regions, while the APRs for rectal cancer were more common in lower-volume regions. These findings are consistent with those of Ricciardi et al., who found a large variation in the county-level rates of non-restorative techniques for rectal cancer ranging from 5.9% to 100% [15,16]. Moreover, they found that 38.8% of surgeons that treated rectal cancer did not perform a restorative reconstruction [15,16]. Our study built on the work of Riccardi et al., which included hospital discharge data for only 11 states, by including the entirety of the United States in 2014. In addition, our study was the first to include the influence of the colon and rectal surgeon density using data obtained by the American Board of Colon and Rectal Surgery.

Our findings detail the existence of regional variation in the surgical approaches, a worrisome disparity for Medicare beneficiaries across the United States, as the eligibility for restorative reconstruction appears to depend in part on the region in which a patient chooses care and not solely on clinical characteristics. Previous work showed the presence of an ostomy for rectal cancer survivors is associated with a lower quality of life, worse illness perceptions, and higher healthcare consumption when compared with non-ostomy survivors [17,18,19,20,21]. Thus, this disparity has large population health effects in areas with a higher utilization of APRs than expected. This disparity is further magnified should the patient choose care in a lower-volume region, where the surgical care team may not be experienced in the perioperative care of rectal cancer, leading to increased complication rates and worse oncologic outcomes [22]. Recent work confirmed this volume–outcome relationship for rectal cancer surgery regardless of specialty, with the effect more pronounced in the United States than in the rest of the world [22,23]. On a national level, this translates to increased healthcare utilization and Medicare expenditures for suboptimal care. As this double-hit disparity appears to exist in rectal cancer, it may follow that similar disparities exist in other colorectal disease treatment, compounding these effects.

A higher regional concentration of colon and rectal surgeons was associated with higher rates of LARs and lower rates of APRs. Although factors affecting the eligibility of a restorative procedure (stage, tumor distance from anal verge) may differ between individual patients, these were likely modest differences at the hospital referral regional level of 120,000 to 8,000,000 Medicare beneficiaries, where individual clinical differences could not wholly explain this observation. Colon and rectal surgeons are trained to perform complex pelvic surgery, and naturally a higher regional concentration would result in greater rates of restorative reconstruction and lower rates of permanent colostomy formation.

We also found a lower intragroup variation in LAR rates with higher concentrations of colon and rectal surgeons, suggesting rectal cancer patients may have been more likely to be treated by surgeons with a more homogenous practice pattern—in this case, colon and rectal surgery specialists. Longstanding patterns of referral to non-colorectal surgeons performing rectal cancer surgery likely have a greater influence on the regional variation in regions with a low colon and rectal surgeon density over high-density ones. Another reason for the lower intragroup variation may have been due to the development of a “regional standard for rectal cancer treatment” among all the surgeons treating rectal cancer, both colon and rectal and non-colon-and-rectal surgeons, with an increasing colon and rectal surgeon density [24,25,26].

The higher regional density of colon and rectal surgeons likely influenced both the volume of major rectal cancer cases performed and the resultant outcomes. This was strongly due to the specific surgical training that occurs in colon and rectal surgery residency. This training allows for the use of minimally invasive surgery, ERAS protocols, and high-quality ostomy creation and care. In addition, a higher density of colon and rectal surgeons is more likely to facilitate the development of multidisciplinary rectal cancer teams and tumor boards. The development of these teams further improves rectal cancer care by bringing other rectal cancer specialists, such as gastrointestinal oncologists, radiation oncologists, gastrointestinal radiologists, and gastrointestinal pathologists, into hospital referral regions and by creating a multidisciplinary treatment plan for rectal cancer that is consistent with the contemporary standard of care. Potential solutions to allow for a higher volume and better outcomes with rectal cancer surgery and care include hospital referral region incentives for the development of rectal cancer care teams in low-density HRRs, minimum volume standards by Medicare for major rectal cancer surgery, and/or increasing surgical training spots in colon and rectal surgery.

Our findings reflect the trends toward increasing the rates of restorative reconstruction after a proctectomy for rectal cancer and the regionalization of cancer care to specialized centers [1]. The American College of Surgeons National Accreditation Program for Rectal Cancer (ACS-NAPRC), a collaboration between the Consortium for Optimizing the Treatment of Rectal Cancer (OSTRICh) and the Commission on Cancer, is a quality program aimed at improving the quality of rectal cancer care through standardized structural and process measures and iterative improvement [27]. These efforts are largely led by colon and rectal surgeon specialists. Through this accreditation process, we may find that there is further movement to the regionalization of rectal cancer care to ACS-NAPRC-accredited centers through either patient choice or insurance coverage limitations.

This study was limited by its analysis of only Medicare claims data, which captures a large proportion of the U.S. rectal cancer cases given the typical advanced age of onset for patients with the disease, but it is not complete. Furthermore, data suppression in HRRs with less than 11 APRs or LARs in 2014 excluded areas of low population density, leaving an incomplete characterization of all the Medicare patients with rectal cancer that underwent surgical intervention in 2014. And these data lack the rates of outpatient neoadjuvant therapy and patient/tumor specific characteristics. This was of greater significance in our APR analysis, as the LARs were more common overall and tended to be performed in regions of greater rectal cancer surgical volumes. In addition, there has been further development in rectal cancer treatment, including total neoadjuvant therapy, radiation-sparing techniques, and immunotherapy, which were not the standard of care in 2014. Our data do not capture the rates of restorative or non-restorative resections on an individual surgeon level, and thus, we can only infer the association. We were limited to colon and rectal surgeons per MB without consideration of the density per total general surgeons, and as such, our conclusions were limited to the correlation of the observed trends.

In conclusion, a higher regional concentration of board-certified colon and rectal specialists was associated with higher rates of LARs and lower rates of APRs in Medicare beneficiaries that underwent surgery for rectal cancer. Future work should explore this association, including the effects of the total surgeon density, specialty types, and temporal variation within each region on these rates to further test these findings.

## Data Availability

The original contributions presented in this study are included in the article. Further inquiries can be directed to the corresponding author.

## Abbreviations

ABCRS	American Board of Colon and Rectal Surgery
APR	Abdominoperineal resection
ARG	Atlas Rate Generator
HRR	Hospital referral region
LAR	Low anterior resection
MB	Medicare beneficiary
